# Supplementary material for: Shiny-MAGEC: A Bayesian R shiny application for meta-analysis of censored adverse events
Source: Res Synth Methods. 2025 Nov 24;17(2):378–88. doi: 10.1017/rsm.2025.10052 (PMC12873611; doi:10.1017/rsm.2025.10052)
Supplement: Zhou et al. supplementary material [file S1759287925100525sup001.pdf]

Supplementary material for "Shiny-MAGEC: A Bayesian R Shiny Application for Meta-analysis of Censored Adverse Events" by Zhou et al.

Web Appendix. Sample JAGS Model Code

```
model {
  for (i in 1:(J1+J2)) {
    logit(p[i]) <- u[study[i]]
  }

  # Case 1: fully-observed
  for (i in 1:J1) {
    Y[i] ~ dbin(p[i], N[i])
  }

  # Case 2.1: Left-censored
  for (i in 1:J2){
    Z[i] ~ dbern(cumu[i])  #Z=1
    cumu[i] <- pbin(cutoff_L[J1+i], p[J1+i], N[J1+i])
  }

  # Case 2.2: Right-censored
  # P(Y>=cut_R)=1-P(Y<=cut_R-1)
  for (i in 1:J2){
    Z[i] ~ dbern(cumu[i])  #Z=1
    cumu[i] <- 1 - pbin(cutoff_R[J1+i]-1, p[J1+i], N[J1+i])
  }

  # Case 2.3: Interval-censored
  # P(cut_R <= Y <= cut_L) = P(Y <= cut_L) - P(Y <= cut_R-1)
  for (i in 1:J2){
    Z[i] ~ dbern(cumu[i])  #Z=1
    cumu[i] <- pbin(cutoff_L[J1+i], p[J1+i], N[J1+i]) - pbin(cutoff_R[J1+i]-1, p[J1+i], N
    [J1+i])
  }

  # Prior specifications
  tau ~ dt(0, 1/A^2, 1)T(0,)  #A=2.5 (or 10, 25)
  mu ~ dnorm(0, 1/tau0^2)  #tau0=100

  for (i in 1:n_study) {
    u[i] ~ dnorm(mu, pow(tau, -2))
  }

  # Generated quantities
  # 1) Overall AE incidence probability
```

```
  p_ov <- ilogit(mu)

  # 2) Predicted Interval
  u_new ~ dnorm(mu, pow(tau,-2))
  p_pred <- ilogit(u_new)

  # 3) Study-specific incidence prob.
  for (i in 1:n_study){
    p_study[i] <- ilogit(u[i])
  }

}
```
